# Determinants of the utilization of postpartum family visits: Evidence from rural areas of Eastern China

**DOI:** 10.1371/journal.pone.0194061

**Published:** 2018-03-22

**Authors:** Hai Gu, Hua You, Zhiwen Yan, Nichao Yang, Yun Kou, Jun Sun, Ting Yu, Ning Zhang

**Affiliations:** 1 Center for Health Policy and Management Studies, Nanjing University, Nanjing, China; 2 Department of Social Medicine and Health Education, School of Public Health, Nanjing Medical University, Nanjing, China; 3 The First Clinical Medical School, Nanjing Medical University, Nanjing, China; 4 Mayor's Office, Yangzhou City People’s Government, Yangzhou, China; 5 Department of Health Policy and Promotion, School of Public Health and Health Sciences, University of Massachusetts Amherst, Amherst, Massachusetts, United States of America; National Academy of Medical Sciences, NEPAL

## Abstract

**Background:**

Postpartum Family Visits (PFVs) have been advocated as a way to improve health outcomes for puerperal women and their newborns. This study aimed to identify individual factors associated with the utilization of PFVs in rural Jiangsu Province, China.

**Methods:**

We employed responses of the household survey in Jiangsu province, part of the National Health Service Survey (NHSS), a nationally representative survey in China. The data analysis framework was designed based on Andersen’s behavioral model. The outcome variables included nonuse and deficient use of PFVs, and the explanatory variables were organized into three hierarchical levels: predisposing, enabling and need factors. Univariate analysis and multivariate logistic regression analysis were conducted to examine the impact of the three hierarchical levels on PFVs utilization.

**Results:**

A total of 884 rural women who had a childbearing history in the prior five years answered PFVs questions. About 23.4% of them had never received any PFVs, and 40.4% received <3 visits. In the results of multivariate logistic regression, educational level (OR = 0.43, 95% CI: 0.24–0.77), income (OR = 0.62, 95% CI: 0.43–0.88), the distance from the nearest hospital (OR = 1.49, 95% CI: 1.07–2.07) and parity (OR = 2.17, 95% CI: 1.54–3.05) had significant relationship with nonuse. Factors significantly associated with deficient use of PFVs included employment (OR = 0.62, 95% CI: 0.39–0.98), the distance from the nearest hospital (OR = 1.73, 95% CI: 1.26–2.36), level of delivery institution (OR = 1.57, 95% CI: 1.14–2.17), and parity (OR = 1.45, 95% CI: 1.03–2.05).

**Conclusion:**

The study found lower Social Economic Status (SES), long distance with primary health institutions, and the increased need for services stemming from multi-parity reduced the likelihood of nonuse or deficient use of PFVs in rural areas. Multiparous, low SES women and those living far away from primary health institutions should be paid more attention to assure the coverage of postpartum care.

## Introduction

The postpartum period is a time of transition for a woman and her new family, when adjustments need to be made on physical, psychological and social levels [1]. Postpartum hospital stays for a vaginal birth in North America are often less than 48 hours for a vaginal birth, and 72–96 hours in China [2]. Thus most postpartum care is provided in the community and in ambulatory settings. Guidelines from the United States and the World Health Organization emphasize the importance of early follow-up from an experienced clinician to prevent infant and maternal morbidity, after hospital discharge for women and their families [2,3].

Studies have demonstrated that timely and adequate postpartum family visits (PFVs) are positively related to successes of early diagnoses, and prevention of medical complexities among puerperal women and their newborns [4]. Despite of the importance and benefits to women and infants, low utilization of postpartum care continues to be a health concern among low-income women and in developing areas [5–10].

World Health Organization (WHO) and the United Nations Children’s Fund (UNICEF) guidelines recommend a postnatal care visit for the mother and her newborn on day 1, day 3, and day 7 after birth, with continuing contact throughout the first six weeks of life [11]. In China, PFVs refer to professional checks provided by community health workers within six weeks after childbirth [12]. These visits are an important element in the postpartum care, as well as in the systematic care management of pregnant women. The government’s target goal is that at least 70% of urban women and 60% of rural women receive at least three health-care provided PFVs within six weeks after delivery [13]. While there are high rates of PFVs in some developed cities and regions in China, PFVs rates are not satisfactory in many other areas [14–17]. In addition, the PFV rate in rural areas is relatively low. The third National Health Service Survey (NHSS) showed that the rate of women having at least one PFV was 52% in rural areas and only 37% in the poorest rural areas [18]. A research in 2013 also disclosed the low rate of use of PFVs among rural mothers [19]. Hence, improving PFVs nationwide, particularly in rural area as a means to improve maternal and newborn health has become one of the essential initiatives to promote rural health in China.

Various studies have found that the level of postpartum health is highly correlated with the level of economic development, i.e., higher in eastern coastal areas, the most developed area in China, than the central and western (underdeveloped) regions. However, some evidences proved that, with the generally sufficient and satisfactory maternal health services supply, the PFVs still needs to be improved in the rural area in Eastern China [20].

Previous studies have focused on identifying provider-specific factors that correlated the low utilization of PFVs in rural areas [21, 22]. There has been, however, paucity of studies that examined woman-specific characteristics that may be related to nonuse and the deficient use of PFVs [14]. In Eastern China, there is generally sufficient maternal health services providers, and the quality of such care is usually satisfactory. Hence, individual-level factors may contribute greatly to inadequate use of PFVs.

Andersen has described and revised a model of health services utilization that has proved to be a valuable evaluation tool in many settings [22–24]. But it was rarely applied to explain the utilization behaviors of postpartum health services. Postpartum Family Visits were the major service mode for postnatal women and also one of the most important postpartum health services, though this study used PFVs utilization as the research outcome of health services utilization.

Using Anderson’s Behavioral Model of Health Care Utilization as a template, this study aims to address this important question and identify patient-level determinants associated with PFVs utilization among rural women in east coast China.

## Materials and methods

### Conceptual model

The Anderson’s Model was applied in this study to examine the individuals’ factors that are related to the utilization of PFVs. According to the model, usage of health is determined by three dynamics: predisposition to use services, factors which enable or impede use, and their need for care [25, 26].

In our study, predisposing factors included demographic variables and socioeconomic status that are prior to demand for PFVs, including age, marital status, educational level and employment status [27–29]. Enabling factors referred to the logistical aspects of obtaining care, such as individual income, and access to PFV providers. In this study, access to PFV providers was measured by distance between home residency and the nearest hospital that provided PFVs, and the level of the delivery hospitals. It is worth noting that all Chinese hospitals are organized according to a 3-tier system on a basis of a hospital's ability to provide medical care, medical education, and conduct medical research. According to this standard, hospitals are designated as primary, secondary or tertiary institutions [30]. And finally, need factors, which could account for the most immediate reasons for PFV use, included the individual’s perceived health care need and other indicators of their health status. In this study, women’s chronic conditions, the mode for the most recent delivery and parity were included as measures of need factors. Subjects were divided into two groups (having or not having chronic diseases) based on whether they had been diagnosed with high blood pressure, diabetes or other chronic diseases in the last year. Delivery mode was divided into vaginal and cesarean sections. And parity was divided into primi-parity and multi-parity.

### Data and sampling

This paper employed the data on utilization of PFVs in rural area of Jiangsu Province from the National Health Services Survey (J-NHSS), which has been organized by the National Health and Family Planning Commission (NHFPC) every 5 year since 1993[18]. This was the fifth survey and conducted in 2013. In rural area, The J-NHSS covered 11 counties, and the investigation sites were geographically distributed in five counties in the south, three in the middle, and three in the north.

A multi-stage stratified cluster random sampling technique was used to choose the sample in Jiangsu. First, the counties were chosen randomly. Second, towns were drawn from each county, and then villages were drawn randomly from each town. Finally, the number of households drawn from each village was determined according to the proportion of the number of households [31]. When the survey on the same household failed three times (Most of the reason was that no one was home.), the investigators gave up this household, surveyed the replacement from the eligible candidates in order, and ensured that the sample size could meet the requirements for the NHSS project preset. Finally, the completion rate was 95%, and all those households invited responded the questionnaires.

The investigators were local medical personnel (i.e., nurses and doctors) and recruited college students trained by researchers from the medical university. After written consent forms were explained and accepted, all members of a household were interviewed individually at home by one of the investigators. The questionnaires were checked and quality was controlled by survey supervisors who were professionals from the local county centers for disease prevention and control (CDC) and university. Ethical approval was obtained from the Medical Faculty Ethics Committee of Nanjing Medical University.

The study subjects were women with childbearing history in the past 5 years in rural Jiangsu. Our final sample included 896 women.

### Data analysis

The dependent variables included two dichotomous variables indicating the utilization of PFVs. The first one was whether a woman never utilized PFVs ever during six weeks after delivery. The second one was the occurrence of deficient use among all women who used PFVs, measured by the situation that a woman used less than three times (<3) PFVs since a minimum three visits are required. Thus, the participants were firstly divided into nonuse group and use group; then those who used PFVs were divided into two groups according to the required times (≥3 or <3).

According to Andersen’s Model [24, 26], all independent variables were categorized into three groups: predisposing characteristics, the enabling factors, and the need for PFVs. Univariate analyses were firstly applied with Chi-square tests for dichotomous variables. Multivariate logistic regression analyses were used to estimate the odds ratio (OR) for PFVs utilization based on predictor variables, and to examine the factors that were associated with nonuse of PFVs, and deficient of PFVs, in two different models. In the logistic regression analyses, the degree of fit for each model was verified through the Hosmer-Lemeshow (H-L) test.

All data analyses were performed using SPSS version 20.0 (IBM, New York, New York, USA), and the significance level was set at 0.05.

## Results

### Characteristics and PFVs utilization of study population

As shown in Table 1, there were a total of 896 rural women with childbearing history in the five years prior to the survey. Among them, 884 women had valid answers to questions related to PFVs. The valid response rate was 98.7%.

**Table 1:** Characteristics of the rural puerperal women.

Variables	All Women (N = 896)	Women with PFVs answers (N = 884)
Postpartum family visit		
No use (%)		23.4
<3 (%)		40.4
Age (mean*±*SD)	27.7±3.6	27.7±3.6
>27 (%)	44.3	44.1
Married (%)	87.8	87.9
High school and above (%)	13.6	13.5
Employment at survey (%)	84.5	84.6
Income (mean*±*SD)	17077.6±13896.9	17051.5±13937.8
>16,000 (%)	38.6	38.2
Missing (%)	0.3	0.3
Distance between residence and the nearest hospital was1km or more (%)	50.4	50.5
Delivery institution (%)		
Primary	39.6	39.8
Secondary and above	59.5	59.8
Missing	0.9	0.6
Having chronic diseases (%)	27.9	27.6
Delivery mode (%)		
Vaginal	46.5	46.8
Caesarean	53.2	53.2
Missing	0.3	
Parity(%)		
Primiparous	46.5	63.1
Multiparous	53.3	36.9
Missing	0.2	

27 was a mean of age.

Income: annual per capital income, and 16000 Yuan was a median of income

Chronic diseases: Hypertension, diabetes or any other chronic diseases diagnosed by a doctor.

Table 1 represented the utilization of PFVs in our study sample. Among the 884 women who had valid reports, 23.4% of them had never received any postpartum visit (nonuse of PFVs), 40.4% received less than three visits (deficient use of PFVs), and only 36.2% received three or more visits. Among the 884 women, the mean age was 27.7 (SD = 3.6), the average family income per person varied from 833 to 175,000 Yuan (RMB) with a median of 16,000 Yuan, and 787 (87.9%) were married.

### Determinants associated with nonuse of PFVs

Table 2 showed the association between three groups of risk factors and nonuse of PFVs. From the univariate analysis, predisposing characteristics, i.e., married women (OR = 2.01, 95%CI: 1.14–3.56), women with high school diploma or higher (OR = 0.43, 95%CI: 0.25–0.76), and those who were employed (OR = 1.93, 95%CI: 1.17–3.20) were all statistically correlated with nonuse of PFV. In terms of enabling resources, income (OR = 0.53, 95%CI: 0.38–0.75) and the distance from the nearest hospital (OR = 1.45, 95%CI: 1.06–1.98) were statistically correlated with nonuse of PFVs. In terms of the need for health services, only parity (OR = 2.30, 95%CI: 1.67–3.75) was found to be statistically correlated with nonuse of PFVs.

**Table 2:** Factors associated with nonuse of postpartum family visits.

Variable	n	%	ORu(95%CI)	ORm(95%CI)
***Individuals’ predisposing characteristics***				
Age(n = 884)				
≤27	122	24.7	1	1
>27	85	21.8	0.85(0.62,1.17)	0.73(0.52,1.03)
Marital status (n = 884)				
Not married	15	14.0	1	1
Married	192	24.7	2.01(1.14,3.56)*	1.73 (0.94,3.19)
Education(n = 884)				
Below high school	192	25.1	1	1
High school and above	15	12.6	0.43(0.25,0.76)**	0.43(0.24,0.77)**
Employed(n = 884)				
No	20	14.7	1	1
Yes	187	25.0	1.93(1.17,3.20)**	1.67(0.97,2.89)
***Enabling resources***				
Income(n = 881)				
≤16000	148	27.3	1	1
>16000	56	16.6	0.53(0.38,0.75)***	0.62(0.43,0.88)**
The distance from the nearest hospital (n = 884)				
Less than 1km	88	20.1	1	1
1 km or more	119	26.7	1.45(1.06,1.98)*	1.49(1.07,2.07)*
Delivery institution(n = 879)				
Primary	80	22.9	1	1
Secondary and above	122	23.1	1.01(0.73,1.40)	1.22(0.87,1.71)
***The need for health services***				
Chronic diseases (n = 884)				
No	156	24.4	1	1
Yes	51	20.9	0.82(0.57,1.17)	0.94(0.64,1.39)
Delivery mode (n = 884)				
Vaginal	100	24.2	1	1
Caesarean	107	22.8	0.93(0.68,1.26)	1.01(0.73,1.42)
Parity(n = 883)				
Primiparous	99	17.7	1	1
Multiparous	108	33.1	2.30(1.67,3.15)***	2.17(1.54,3.05)***

ORu: the odds ratio of univariate logistic regression analysis; ORm: the odds ratio of multivariate logistic regression analysis.

n = 876 (99.1% of 884) after exclusion of missing data for all covariates in multivariate analysis.

* p<0.05

** p<0.01

*** p<0.001

With controlling for various variables, the multivariate logistic analysis had similar patterns but significance level changed. Among predisposing factors, only educational level had significant relationship with nonuse of PFVs: women who graduated from high school were 57% (OR = 0.43, 95%CI: 0.24–0.77) less likely to have no experience in PFV use compared to those who were high school dropouts. Other predisposing factors had the same direction as in univariate analysis but with no statistical significance. For enabling factors, i.e., women whose family income being at least 16,000 a year, were 38% (OR = 0.62, 95%CI: 0.43–0.88) less likely to never use PFVs; women whose residence was farther than one kilometers from a nearest hospital were 49% (OR = 1.49, 95%CI: 1.07–2.07) more likely not to use PFVs; and women who delivered their babies in secondary or tertiary hospitals were 22% (OR = 1.22, 95%CI: 0.87–1.71) more likely not to use PFVs, although this results were statistically insignificant. Among the factors related to need for PFVs, women with multiparous births were more than doubled the likelihood of not using PFVs, compared to those with primiparous births (OR = 2.17, 95%CI: 1.54–3.05). The Hosmer-Lemeshow (H-L) test showed good model degree of fit (P = 0.45) [32].

### Determinants associated with deficient use of PFVs

We further analyzed the factors associated with deficient use (i.e., <3 visits) of PFVs among the 677 women who received PFV, as shown in Table 3. According to the results of the univariate analysis, the three indicators that showed statistical correlation with deficient use of PFVs were employment (predisposing factor, OR = 0.63, 95%CI: 0.42–0.95), the distance from the nearest hospital (enabling factor, OR = 1.76, 95%CI: 1.29–2.38), and the delivery institution (enabling factor, OR = 1.66,95%CI: 1.22–2.26). No need factors of PFVs had significant relationship with deficient use of PFVs. Multivariate analysis showed the similar trend. The OR(95%CI) of employment, the distance from the nearest hospital, delivery institution, and parity were 0.62(0.39,0.98), 1.73(1.26,2.36), 1.57(1.14,2.17), and 1.45(1.03,2.05), respectively. In multivariate logistic regression analysis, the H-L test showed good model degree of fit (P = 0.90).

**Table 3:** Factors associated with deficient use of postpartum family visits.

Variable	n	%	ORu(95%CI)	ORm(95%CI)
***Individuals’ predisposing characteristics***				
Age(n = 677)				
≤27	185	49.7	1	1
>27	172	56.4	1.31(0.96,1.77)	1.22(0.89,1.68)
Marital status (n = 677)				
Not married	50	54.3	1	1
Married	307	52.5	0.93(0.60,1.44)	1.08(0.66,1.76)
Education(n = 677)				
Below high school	294	51.3	1	1
High school and above	63	60.6	1.46(0.95,2.23)	1.44(0.93,2.25)
Employed(n = 677)				
No	72	62.1	1	1
Yes	285	50.8	0.63 (0.42,0.95)*	0.62(0.39,0.98)**
***Enabling resources***				
Income(n = 677)				
≤16000	202	51.1	1	1
>16000	155	55.0	1.17(0.86,1.58)	1.19(0.86,1.65)
The distance from the nearest hospital (n = 677)				
Less than 1km	161	46.0	1	1
1 km or more	196	59.9	1.76(1.29,2.38)***	1.73(1.26,2.36)***
Delivery institution(n = 677)				
Primary	122	45.2	1	1
Secondary and above	235	57.7	1.66(1.22,2.26)***	1.57(1.14,2.17)**
***The need for health services***				
Chronic diseases (n = 677)				
No	254	52.5	1	1
Yes	103	53.4	1.04(0.74,1.45)	1.01(0.71,1.44)
Delivery mode (n = 677)				
Vaginal	170	54.1	1	1
Caesarean	187	51.5	0.90(0.67,1.22)	0.84(0.61,1.16)
Parity (n = 677)				
Primiparous	231	50.3	1	1
Multiparous	126	57.8	1.35(0.98,1.87)	1.45(1.03,2.05)*

ORu: the odds ratio of univariate logistic regression analysis; ORm: the odds ratio of multivariate logistic regression analysis.

n = 876 (99.1% of 884) after exclusion of missing data for all covariates in multivariate analysis.

* p<0.05

** p<0.01

*** p<0.001

## Discussion

This study revealed that even in the rural area in Jiangsu province, one of the most developed provinces nationwide, the rate of nonuse of PFVs was about 20% and the rate of deficient use was about 40%, which were both higher than the targets the government has established. Using Andersen’s Behavioral Model of Healthcare Utilization, this paper aimed to examine individual characteristics that are associated with nonuse or deficient use of PFVs in rural China [22]. Our results showed that lower education level and unemployed status (predisposing risk factors), lower income, large distance from hospitals and the second-level and higher delivery institutions (enabling resources), and multiple births (need factor) were positively related to nonuse of PFV services, and unemployment, large distance from hospitals and the second-level and higher delivery institutions were positively related to deficient of PFVs.

Findings from this study are partly consistent with those of previous studies. A study by Lu found that women with low education levels and low income were less likely to utilize postpartum care services compared to their counterparts [33]. Another study by Zhang using a Chinese sampling population found that the PFVs rate in multiparas was lower than that in primiparous [34]. In another study in the eastern provinces of China, Tao discovered that traffic was one of the factors that hindered PFVs [21].

It is widely accepted that education level, occupation, and income are reflective of a person’s social and economic status or SES [35, 36]. Lower education level, unemployment, and lower income are all closely related to lower SES [37]. Results from this study indicate that even in economically developed coastal areas in eastern China, if rural women were situated in lower social strata, they would experience in lower utilization of health care services during pregnancy and childbirth. Hence, poor SES is a risk factor for low utilization of postpartum visits for rural women.

Previous studies have shown that compared to urban areas, medical resources of primary hospitals in rural areas were relatively insufficient and the quality of health services in the rural primary hospitals was quite poor [21, 38]. Hence, whenever is possible, women in rural areas may seek prenatal care and delivery their babies in the secondary or tertiary hospitals nearby in order to receive better care. Nevertheless, according to specific Chinese PFV policies, women who delivered babies in secondary or higher institutions should be transferred to a corresponding community-based, primary institution for neonatal care, including PFVs. In other words, all of PFVs, no matter where mothers delivered their babies, should be provided by the personnel from community primary medical hospitals. Our study found that deficient use of PFVs happens more frequently among women who deliver a baby in secondary or tertiary hospitals than among those in primary hospitals. The lack of or delay in communication during transferring between these hospitals may contribute to this phenomenon. Most secondary or tertiary hospitals have implemented electronic medical record (EMR) system, while many of rural, primary hospitals may have none, or less comprehensive EMR systems. When women delivered their babies in the secondary or tertiary hospitals, it is possible that these hospitals may not provide paper-style patient record timely to community-based primary hospitals at transfer, or cannot share full patients’ information because of incompatible information systems. Whichever is the case, delaying and/or inadequate PFVs provided by the community-based, primary hospitals may occur. Therefore, building up seamless information systems between different levels of hospitals can help to remove the communication block between primary and secondary and above hospitals during transfers [39]. Furthermore, the referral mechanism between the different level medical institutions has been working poorly, particularly referrals from higher level institutions to lower level (i.e., downward referral) institutions [40]. This is mainly because the medical resources are not logically distributed evenly, giving rise to limited resources and poor medical standards in primary institutions. Therefore, there is a great need of establishing a stable and effective referral mechanism, as well as of having a new recommendation that nurture the capabilities of the primary medical institutions in remote rural areas, strengthening the management and supervision of referrals between medical institutions of different levels.

In addition, maternal parity has been mentioned in prior studies as a factor influencing the utilization of health care services during the pregnant and puerperal period [21, 34]. Results from our study demonstrated that multiparous mothers were more likely to experience nonuse or deficient use of PFVs than primiparous mothers. This result is consistent with others that indicate postpartum care receives more attention at the first birth than at subsequent births [41]. In particular, a multiparous woman may believe they have enough experiences, knowledge, and skills of “doing-the-month” (a Chinese traditional calling for the convalescence during the postpartum period). When delivering the second child, women may not pay as much attention to PFVs as they do during their first delivery. Thus, multiparity is another risk factor that may lead to nonuse or deficient use of PFVs. China is now actively reforming its population policy and allowing two children for every couple [42]. Given this social background, this potential risk factor should receive more attention. The second child policy will give rise to a rapid increase in the proportion of multiparous women that will pose new challenges to PFVs and other health care services during the pregnancy and puerperal period.

### Limitations

There are some limitations in this paper. Firstly, ignoring complex survey design may result in bias and low P value. To ensure the development of appropriate estimates, survey design analyses with the sampling weight were suggested to adopt [43]. Due to unavailable sample weights of data of National Health Services Survey, ordinary statistical analyses were still used in this research, and thus the bias may have been unavoidable. Secondly, the NHSS in China was not specifically designed for investigations of pregnant and puerperal women’s health care. Hence, factors like previous abortion history, accidental factors like prolonged labor, and the knowledge and psychological factors of the mothers which also might possibly influence postpartum visits, were not included in our study. In addition, since this study focused on the situations of health care during the pregnant and puerperal period within the five years prior to the survey, memory bias may have occurred [44].

## Conclusions

In spite of limitations, findings from this study contributed to the understanding of the risk factors of nonuse and deficient use of PFVs. The lower social and economic status of women in rural areas, living far from any primary medical care unit in the remote rural areas, and the increased need for services stemming from multi-parity all pose a threat to nonuse or deficient use of PFVs in rural areas.

According to the outcomes of this study, related policies and suggested responses are to strengthen the service capabilities of the primary medical institutions for people living in remote rural areas, and to improve the referral mechanism between institutions of different levels. Special importance should be attached to poor women in remote rural areas; accessibility to maternal and child health care resources should be improved. Considering the changing birth control policy, precautions should be taken to prevent the decrease in health care utilization during the pregnant and puerperal period by the “second child” mothers.

## Supporting information

S1 Dataset (SAV)
